# Rapid detection of cefiderocol susceptibility/resistance in *Acinetobacter baumannii*

**DOI:** 10.1007/s10096-023-04691-w

**Published:** 2023-11-01

**Authors:** Otávio Hallal Ferreira Raro, Maxime Bouvier, Auriane Kerbol, Jean-Winoc Decousser, Laurent Poirel, Patrice Nordmann

**Affiliations:** 1 Medical and Molecular Microbiology, Faculty of Science and Medicine, University of Fribourg, Chemin du Musée 18, CH-1700 Fribourg, Switzerland; 2 Swiss National Reference Center for Emerging Antibiotic Resistance (NARA), University of Fribourg, Fribourg, Switzerland; 3 Department of Bacteriology and Infection Control, University Hospital Henri Mondor, Assistance Publique - Hôpitaux de Paris, Créteil, France; 4 Faculté de Médecine de Créteil, Ecole Nationale Vétérinaire d’Alfort (EnvA), EA 7380 Dynamyc Université Paris-Est Créteil (UPEC), Créteil, France; 5 Institute for Microbiology, Lausanne University Hospital and University of Lausanne, Lausanne, Switzerland

**Keywords:** *Acinetobacter baumannii*, Cefiderocol, Resistance, Rapid test

## Abstract

**Purpose:**

Due to its ability to disseminate worldwide and its multiple resistance trait, *Acinetobacter baumannii* is becoming a threat for public health worldwide. Cefiderocol (FDC) is a promising broad-spectrum cephalosporin recently approved for treating Gram-negative infection. The aim of this study was to develop a rapid test, namely the rapid FDC *Acinetobacter baumannii* NP test, for the detection of FDC susceptibility/resistance in *A. baumannii* since the current FDC susceptibility tests are rather time-consuming (at least 24 h).

**Materials and methods:**

The rapid test is based on the reduction of resazurin to resorufin product by bacterial viable cells, thus detecting bacterial growth in the presence of FDC (38.4 mg/L). A color change from blue (resazurin) to violet or pink (resorufin) represents visual detection of bacterial growth. 95 randomly selected *A. baumannii* isolates were used to evaluate the performance of the rapid FDC *Acinetobacter baumannii* NP test.

**Results:**

The test showed 95.5% (95% CI 78.2–99.2%) and 100.0% (95% CI 95.0–100.0%) of sensitivity and specificity, respectively. All the results were obtained within 4 h30–4 h45 incubation time at 35 °C ± 2 °C, saving virtually one day when compared with currently-used antimicrobial susceptibility tests. The test showed only a single very major error, an isolate with a MIC of 8 mg/L.

**Conclusions:**

The rapid FDC *Acinetobacter baumannii* NP test can be a valuable method which is easier and faster to interpret when compared with the gold standard broth microdilution method. The test showed remarkable performances; hence, it may be suitable for implementation in clinical microbiology routine laboratories.

## Introduction

*Acinetobacter baumannii* is a gram-negative opportunistic pathogen responsible for causing a series of healthcare-associated infections that tend to occur in patients hospitalized in intensive care units and includes pneumonia, bloodstream, urinary, and wound infections [1]. Those infections are difficult to treat and are often combined with longer hospitalization and high mortality rates, generating a great concern for public health [2].

Carbapenems are frequently one important choice for treating these infections; however, outbreaks and reports of carbapenem-resistant *A. baumannii* (CRAB) are already disseminated globally [3]. Consequently, in 2017, the World Health Organization (WHO) included CRABs as “critical” in their list of priority pathogens for research and development of new antibiotics [4]. Carbapenem resistance in *A*. *baumannii* has been related to distinct mechanisms such as outer membrane permeability defects (porins), overexpression of genes encoding efflux pumps, and modification of penicillin-binding proteins (PBPs), but mostly through production of acquired carbapenemases [(KPC Ambler class A), IMP, NDM, VIM (class B), and OXA-23, OXA-24/40, OXA-58, and OXA-72 (class D)] and also through the overexpression of the *bla*_OXA-51_-like gene being intrinsic to this species [2].

Among the last treatment options, colistin and polymyxin B usage is frequently associated with acute kidney injury, and, like tigecycline, both have limited penetration in infection sites such as lung and urine [5]. Furthermore, the novel sulbactam-durlobactam combination does not inhibit the strains producing class B metallo-β-lactamases (MBL) [6]. Hence, there is an urgent need to search and develop new drugs for treating *A. baumannii* infections. FDC is a siderophore cephalosporin which is able to bind to extracellular free iron via its siderophore side chain, allowing active transport into the periplasmic space of gram-negative bacteria through siderophore uptake systems, and, in addition, is also able to invade bacterial cells by passive diffusion through outer membrane porin channels [7].

FDC has demonstrated in vitro activity against the majority of β-lactamases, including MBLs, and has been approved by the FDA and EMA, respectively, in 2019 and 2020, for the treatment of infections due to aerobic gram-negative microorganisms in adults with complicated urinary tract infections (cUTI), including pyelonephritis caused by susceptible strains [7, 8]. Although its recent approval for use in humans and its limited usage, isolates of CRAB resistant to FDC have been reported and associated with PER-like β-lactamases [9], NDM-like β-lactamases [9], point mutations in PBP3 and OXA-23 [10], and *piuA* (TonB-dependent siderophore receptor) or *pirA* (siderophore gene) [11].

The broth microdilution (BMD) method, the standard reference for determining susceptibility to *A. baumannii*, is rather time-consuming, and interpretation of the results is quite debatable and challenging for a microbiology routine laboratory [12]. Other antimicrobial susceptibility techniques such as disk diffusion methods (30 μg FDC discs, Liofilchem, Roseto degli Abruzzi, Italy) and UMIC® Cefiderocol (Bruker Daltonics, Bremen, Germany) can be used as an alternative, but they still require at least 18 h to obtain the results. Moreover, currently, the FDA (susceptible ≤ 1; intermediate: 2; resistant > 4 mg/L) [13], the CLSI (susceptible ≤ 4; intermediate: 8; resistant > 16 mg/L) [14], and the EUCAST (susceptible ≤ 2; resistant > 2 mg/L) [15] have different breakpoints for interpreting FDC susceptibility results for *A. baumannii*, turning its interpretation even more challenging.

Thus, the development of an inexpensive, rapid, precise, and trustful test for the detection of FDC resistance in *A. baumannii* may be beneficial to optimize the treatment of patients infected by *A. baumannii*. The rapid FDC *Acinetobacter baumannii* NP test was therefore designed for that purpose.

## Materials and methods

### *Bacterialisolates and antimicrobial susceptibility testing*

95 *A. baumannii* isolates were randomly selected from the collection of the Swiss National Reference Center of Emerging Antibiotic Resistance (NARA). Isolates from this collection were previously sent to the reference center for investigation of their carbapenemase content. The main β-lactamase gene as a source of the multidrug resistance pattern of all isolates had been previously characterized, and it is shown on Table 1.

**Table 1:** Results of the rapid FDC NP test for detection of cefiderocol susceptibility/resistance in *Acinetobacter baumannii* isolates

Strain number	Main β-lactamase gene	BMD FDC (µg/mL)	FDC phenotype	Rapid FDC test	
				Result	Discrepancies versus BMD
CIP 70.10 (ATCC 15151)	-	0.5	S	-	-
1	OXA-23	≤ 0.125	S	-	-
2	OXA-23	≤ 0.125	S	-	-
3	OXA-23	≤ 0.125	S	-	-
4	OXA-23	≤ 0.125	S	-	-
5	OXA-23	≤ 0.125	S	-	-
6	OXA-23	≤ 0.125	S	-	-
7	OXA-23	0.25	S	-	-
8	OXA-23	0.25	S	-	-
9	OXA-23	0.25	S	-	-
10	OXA-23	0.25	S	-	-
11	OXA-23	0.5	S	-	-
12	OXA-23	0.5	S	-	-
13	OXA-23	0.5	S	-	-
14	OXA-23	0.5	S	-	-
15	OXA-23	0.5	S	-	-
16	OXA-23	0.5	S	-	-
17	OXA-23	0.5	S	-	-
18	OXA-23	0.5	S	-	-
19	OXA-23	0.5	S	-	-
20	OXA-23	0.5	S	-	-
21	OXA-23	0.5	S	-	-
22	OXA-23	0.5	S	-	-
23	OXA-23	0.5	S	-	-
24	OXA-23	0.5	S	-	-
25	OXA-23	0.5	S	-	-
26	OXA-23	0.5	S	-	-
27	OXA-23	0.5	S	-	-
28	OXA-23	0.5	S	-	-
29	OXA-23	0.5	S	-	-
30	OXA-23	0.5	S	-	-
31	OXA-23	0.5	S	-	-
32	OXA-23	0.5	S	-	-
33	OXA-23	0.5	S	-	-
34	OXA-23	0.5	S	-	-
35	OXA-23	0.5	S	-	-
36	OXA-23	1	S	-	-
37	OXA-23	1	S	-	-
38	OXA-23	1	S	-	-
39	OXA-23	1	S	-	-
40	OXA-23	1	S	-	-
41	OXA-23	1	S	-	-
42	OXA-23	1	S	-	-
43	OXA-23	1	S	-	-
44	OXA-23	1	S	-	-
45	OXA-23	1	S	-	-
46	OXA-23	1	S	-	-
47	OXA-23	1	S	-	-
48	OXA-23	1	S	-	-
49	OXA-23	1	S	-	-
50	OXA-23	1	S	-	-
51	OXA-23	1	S	-	-
52	OXA-23	1	S	-	-
53	OXA-23	1	S	-	-
54	OXA-23	1	S	-	-
55	OXA-23	1	S	-	-
56	OXA-23	1	S	-	-
57	OXA-23	1	S	-	-
58	OXA-23	1	S	-	-
59	OXA-23	1	S	-	-
60	OXA-23	2	S	-	-
61	OXA-23	2	S	-	-
62	OXA-23	2	S	-	-
63	OXA-23	2	S	-	-
64	OXA-23	2	S	-	-
65	OXA-23	2	S	-	-
66	OXA-23	2	S	-	-
67	OXA-23	2	S	-	-
68	OXA-23	**8**	R	-	VME
69	OXA-40	≤ 0.125	S	-	-
70	OXA-40	≤ 0.125	S	-	-
71	OXA-40	≤ 0.125	S	-	-
72	OXA-58	≤ 0.125	S	-	-
73	OXA-58	≤ 0.125	S	-	-
74	OXA-58	0.5	S	-	-
75	OXA-23	**4**	R	+	-
76	OXA-23	**16**	R	+	-
77	OXA-23	**16**	R	+	-
78	OXA-23	**32**	R	+	-
79	OXA-23	**32**	R	+	-
80	OXA-23	**64**	R	+	-
81	OXA-23	**64**	R	+	-
82	OXA-23	**64**	R	+	-
83	OXA-23	**64**	R	+	-
84	OXA-23	**128**	R	+	-
85	OXA-23	** ≥ 128**	R	+	-
86	OXA-23 + NDM-1	**8**	R	+	-
87	OXA-23 + NDM-5	**64**	R	+	-
88	OXA-26	**64**	R	+	-
89	OXA-40	**64**	R	+	-
90	OXA-58	**64**	R	+	-
91	OXA-72	**64**	R	+	-
92	GES-12	**64**	R	+	-
93	IMP-4	**64**	R	+	-
94	NDM-1	**4**	R	+	-
95	AmpC overproduced	**64**	R	+	-

Bold values are resistant. Normal script values are susceptible

*BMD* broth microdilution, *FDC* cefiderocol, *S* susceptible, *R* resistant,—no discrepancies observed, *VME* very major error

The BMD reference method was performed to determine the minimum inhibitory concentration (MIC) of all isolates following the European Committee on Antimicrobial Susceptibility Testing (EUCAST) guidelines [14]. Results were interpreted according to the pharmacokinetic/pharmacodynamic (PK/PD) breakpoints from EUCAST (susceptible ≤ 2 mg/L; resistant > 2 mg/L) [15, 16]. BMD was considered the gold standard when comparing to the results obtained with the rapid FDC *Acinetobacter baumannii* NP test. The reference strain *A. baumannii* CIP 70.10 (ATCC 15151) was used as quality control for both techniques, and the reference *Pseudomonas aeruginosa* (ATCC 27853) strain was also used for BMD quality control.

#### Optimizing rapid FDC Acinetobacter baumannii NP test

The rapid test is based on the reduction of resazurin (a viability colorant) to resorufin product by bacterial viable cells, thus detecting bacterial growth in the presence of a fixed concentration of FDC. Bacterial growth is visually detected by a color change from blue (resazurin) to violet or pink (resorufin).

In order to reach optimal conditions of the test, several different parameters were tested using the FDC-susceptible *A. baumannii* CIP 70.10 and one resistant isolate with a MIC ≥ 128 mg/L. These parameters comprised variable FDC concentrations (8, 16, 24.8, 32, 38.4, 46.2, 64, and 128 mg/L), variable bacterial inoculum concentrations (0.5 of the McFarland’s scale, 1/5, 1/10, 1/15, 1/20, 1/25 of the 1.0 McFarland’s scale), different bacterial inoculum volumes (20 and 50 µL), different reagent solution volume (150 and 180 µL), and variable incubation times (4 h, 4h15, 4h30, 4h45, and 5 h).

The optimal conditions were FDC final concentration at 38.4 mg/L, inoculum concentration of 1/20 of the 1.0 McFarland’s scale, bacterial inoculum volume of 20 µL, reagent solution volume of 180 µL, and 4h30-4h45 of incubation time.

### Preparation of the rapid FDC Acinetobacter baumannii NP solution

The solution was prepared with iron-depleted Mueller–Hinton broth (ID-MHB; chelex 100 resin, Bio-Rad, Marnes-la-Coquette, France; MHB, AxonLab, Baden, Switzerland) [14] supplemented or not with FDC (Shionogi, Osaka, Japan) at a concentration of 42.67 mg/L targeting to achieve a final fixed concentration of 38.4 mg/L in a 200 µL final volume in each microplate’s well. The solution can be stored at − 80 °C for 2 weeks.

### Bacterial inoculum

Overnight cultures were grown on UriSelect 4 (Bio-Rad, Marnes-la-Coquette, France). After that, a 1.0 McFarland bacterial inoculum was prepared by adding bacterial colonies in 5 mL sterile NaCl (0.85%) and then diluting 1/20 (50 µL of inoculum in 950 µL of NaCl) before inoculation into the microplates in a range from 15 min to 1 h after preparation [15].

### Tray inoculation

**Fig. 1:**
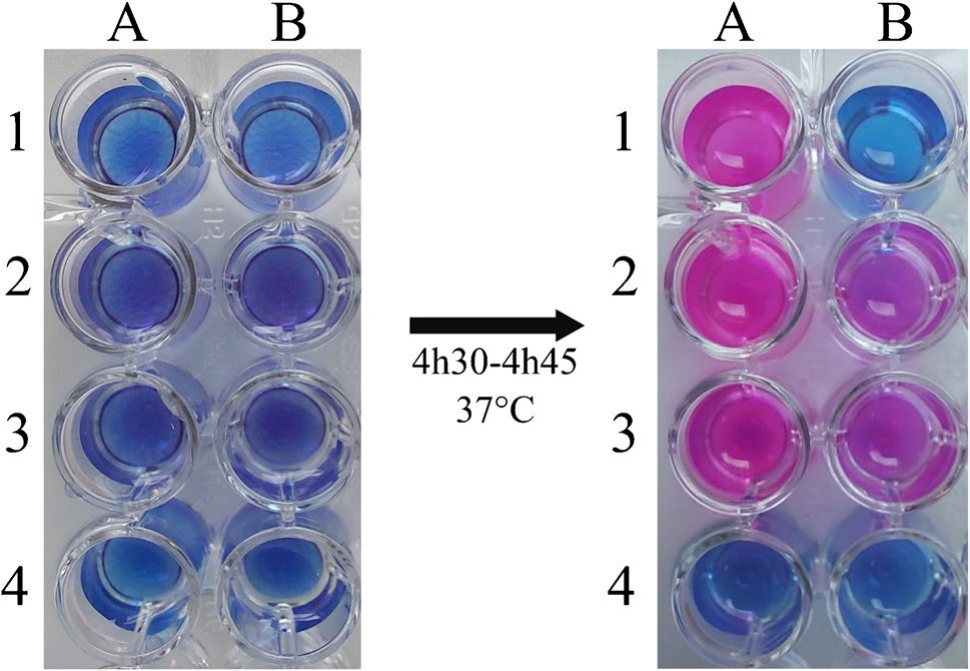
The rapid FDC *Acinetobacter baumannii* NP test. Column A presents the solution free of FDC. Column B presents the solution with FDC (38.4 mg/L). Reference strain *A. baumannii* CIP 70.10 (ATCC 15151) was inoculated in A1 and B1; FDC-resistant isolate (positive control) was inoculated in A2 and B2; tested isolate (resistant to FDC) that grew in both absence and presence of FDC was inoculated in A3 and B3; and NaCl 0.85% was inoculated in A4 and B4 as control of contamination and possible spontaneous color change. Bacterial growth is evidenced by a color change of the medium from blue to pink or violet

A 96-well polystyrene microplate (round base, with lid, sterile; Sarstedt, Nuembrecht, Germany) was used to perform the rapid FDC *Acinetobacter baumannii* NP test. The bacterial suspension was inoculated in two independent wells, with and without FDC. The steps to perform the test were as follows: (1) 180 µL of FDC-free solution was added to wells A1-A4; (2) 180 µL of FDC at a concentration of 42.67 mg/L was added to wells B1-B4; (3) 20 µL (1/20 of the 1.0 McFarland scale) of *A. baumannii* CIP 70.10 (negative control) was added to wells A1 and B1; (4) 20 µL of a FDC-resistant isolate (positive control) was added to wells A2 and B2; (5) 20 µL of a tested isolate was added to wells A3 and B3; and (6) 20 µL of NaCl 0.85% was added to wells A4 and B4 (Fig. 1). After preparing the microplate for the test and before inoculating the bacterial suspensions, the rapid FDC *Acinetobacter baumannii* NP solution was pre-warmed at 37 °C for 15–30 min before use to prevent growth delay and therefore a delayed color change.

### Tray incubation and reading

The tray was not sealed and the test was incubated for 3 h at 35 ± 2 °C without shaking. After that time, 20 µL of resazurin reagent PrestoBlue (Thermo Fisher Scientific, Cleveland, OH, USA) was added to each well [(concentration of 10% (vol/vol)] and the test was incubated again. Reading was performed visually by checking the tray for no spontaneous color change after 4 h and then every 15 min until reaching a total of 4h45 h of incubation. The test was considered positive when the isolate grew (color change from blue to pink or violet) in the presence of FDC, and negative when no growth was observed (no color change) in the presence of FDC. Figure 1 provides a comprehensive illustration of the visual interpretation of the rapid FDC *Acinetobacter baumannii* NP test.

To assure optimal quality control of the test and consequently validate the rapid FDC *Acinetobacter baumannii* NP test, the following conditions had to be reached; (1) blue-to-pink/violet color change observed, confirming the bacterial growth and viability for all isolates in wells without FDC (A1-A3); (2) no color change observed (remaining blue) in wells after adding NaCl 0.85%, confirming the absence of contamination (A4 and B4); (3) blue to pink/violet color changes observed in the wells where the positive control and the tested isolate were added (A2 and B2; A3 and B3); and (4) no color change observed with the reference strain *A. baumannii* CIP 70.10 in the well with FDC (negative control) (B1). See Fig. 1 for some examples.

### Data analysis

Discrepancies between the rapid FDC *Acinetobacter baumannii* NP test and the BMD standard reference method were determined and classified, if present, as very major errors (VME) and major errors (ME) as previously described [17, 18]. Sensitivity, specificity, accuracy, and precision parameters were also calculated to evaluate the performance of the test proposed. Results were blindly read and interpreted independently by two laboratory members.

## Results

The tested collection comprised 95 randomly-selected *A. baumannii* isolates including OXA-23 (*n* = 78 [82.1%]), OXA-24/40 (*n* = 5 [5.26%]), OXA-26 (*n* = 1 [1.05%]), OXA-58 (*n* = 4 [4.2%]), OXA-72 (*n* = 1 [1.05%]), GES-12 (*n* = 1 [1.05%]), IMP-4 (*n* = 1 [1.05%]), NDM-1 (*n* = 1 [1.05%]), OXA-23 co-production with NDM-like (NDM-1 and NDM-5) (*n* = 1 [1.05%] for each one), and AmpC ADC overexpressed (*n* = 1 [1.05%]). A total of 73 (76.8%) of these isolates were susceptible (MIC range: ≤ 0.125 to 2 mg/L) and 22 (23.2%) were resistant (MIC range: 4 to ≥ 128 mg/L) to FDC according to the BMD results and interpreted following the EUCAST guidelines [15]. Those results are summarized in Table 1.

All the FDC-susceptible isolates were negative, and 21 FDC-resistant isolates gave positive results for the rapid FDC *Acinetobacter baumannii* NP test. Only a single resistant isolate with an MIC of FDC at 8 mg/L, being an OXA-23 producer, gave a negative result for the test (Table 1). This was the only VME (false negative) result observed with the test, although no ME (false positive) was identified. The test showed 95.5% (95% CI 78.2–99.2%) of sensitivity, 100.0% (95% CI 95.0–100.0%) of specificity, 98.9% of accuracy, and 100% of precision when compared with the BMD standard method. After incubating for 4 h (1 h after resazurin was added), the color change of the wells was read at every 15 min, and it was concluded that the optimal reading of the final results shall be at 4h30-4h45 after incubation at 35 °C ± 2 °C under an ambient atmosphere.

## Discussion

*A. baumannii*, especially when resistant to carbapenems, is a challenging threat to hospitalized patients once they are able to, not only cause multidrug resistant infections, but also to survive in facility surfaces and shared medical equipment, being a perfect candidate for nosocomial outbreaks. Treating those infections is difficult and frequently ends in failures. The promising FDC molecule is a notable treatment alternative for CRAB infections since it is stable against the hydrolysis activity of ESBLs, class A and D carbapenemases, class C cephalosporinases, and most MBLs [5].

In the present study, an original and accurate test has been developed for the detection of FDC susceptibility/resistance in *A. baumannii* that showed excellent sensitivity and specificity. The rapid FDC *Acinetobacter baumannii* NP test was able to detect FDC susceptibility/resistance within 4h30-4h45, saving approximately 14 h (a day in practice) from the currently available antimicrobial susceptibility tests, including the reference standard BMD. In addition, it is rather easier for interpreting the results, once is only a matter of visually detecting the viable cells growing in cefiderocol by a color change from blue (resazurin) to violet or pink (resorufin), instead of the controversial existence of trailing endpoints that challenges and difficult BMD interpretation for this antibiotic.

The rapid FDC *Acinetobacter baumannii* NP test showed very few discrepancies when compared with the BMD (no ME and only one VME). Moreover, the proposed test revealed a significant correlation with the standard technique in terms of sensitivity, specificity, accuracy, and precision parameters. The only VME identified in this study had an MIC of 8 mg/L and may be related to the slow metabolism of that particular isolate. Of note, a limitation of the present study is that a limited number of FDC-resistant isolates were available to be tested, once this profile remains relatively scarce. Therefore, further testing in other laboratories will be required for assessing the exact performances of the test.

Also notable is that the EUCAST breakpoints were considered for the development of the Rapid FDC *Acinetobacter baumannii* NP test. When the CLSI breakpoints were considered (susceptible ≤ 4; intermediate: 8; resistant > 16 mg/L) [14] and setting intermediate and resistant isolates categorized as positive for the test, the performance of the test remained excellent, with only two ME (false positive, 2.7%, both presenting borderline MICs of 4 mg/L) and only a single VME (false negative, 5.0%, the same isolate that was found to be VME when interpreting using EUCAST), 95.0% of sensitivity (95% CI 76.4–99.1%), 97.3% specificity (95% CI 90.8–99.3%), 96.8% of accuracy, and 90.5% of precision.

The rapid FDC *Acinetobacter baumannii* NP test allows a quick assessment of FDC susceptibility/resistance when testing *A. baumannii* isolates. It nicely complements the rapid Cefiderocol NP test that had been recently developed for assessing the susceptibility/resistance to FDC in Enterobacterales [17]. Additionally, it is rather faster, cheap (± 1 USD per strain), and easier to perform if compared with the gold standard BMD which often results in questionable interpretation.

Following the urgent need for rapid and precise diagnostics in clinical settings, the rapid FDC *Acinetobacter baumannii* NP test was developed in order to provide a valuable method, which is easier and faster to interpret when compared with the recommended standard method. This rapid and inexpensive test can accurately categorize cefiderocol susceptibility/resistance of *A. baumannii* strains. The test showed remarkable sensitivity and specificity parameters; therefore, it may be suitable for implementation in any clinical microbiology laboratory.

## Data Availability

All the data produced for this manuscript will be available upon request to the corresponding author.
